# Review of *Podothrips* from China (Thysanoptera, Phlaeothripidae), with one new species and three new records

**DOI:** 10.3897/zookeys.882.39029

**Published:** 2019-10-23

**Authors:** Li-Hong Dang, Le Zhao, Xia Wang, Ge-Xia Qiao

**Affiliations:** 1 School of Bioscience and Engineering, Shaanxi University of Technology, Hanzhong, 723000, China Shaanxi University of Technology Hanzhong China; 2 Key Laboratory of Zoological Systematics and Evolution, Institute of Zoology, Chinese Academy of Science, No.1 Beichen West Road, Chaoyang District, Beijing 100101, China Institute of Zoology, Chinese Academy of Science Beijing China; 3 College of Life Science, University of Chinese Academy of Sciences, No. 19, Yuquan Road, Shijingshan District, Beijing 100049, China University of Chinese Academy of Sciences Beijing China

**Keywords:** *Podothrips femoralis*, key, taxonomy, Poaceae

## Abstract

*Podothrips* species occur on the leaves of various Poaceae, including bamboo and grasses. An illustrated identification key is given here to the six *Podothrips* species recorded from China. These include *P. femoralis* Dang & Qiao, **sp. nov.**, and *P. sasacola* Kurosawa, *P. odonaspicola* (Kurosawa), and *P. semiflavus* Hood that are newly recorded from China.

## Introduction

Haplothripini, a tribe distributed worldwide, is the only well-defined and named tribe in the Phlaeothripinae (Mound and Minaei 2007). Most species of this tribe are related to flower-feeding, but some are thought to be predatory, such as *Podothrips* species. A list of 34 genera in this tribe was provided by Mound and Minaei (2007) in a review of the species recorded from Australia. Subsequently, Minaei and Mound (2008) revised the Haplothripini from Iran with four genera, and Dang et al. (2014) described 19 genera from Southeast Asia and China in this tribe. Until now, this tribe includes 34 genera and approximately 580 species worldwide.

*Podothrips* species appear to be predators and live on plants of the family Poaceae. The genus is distinguished from other Haplothripini genera by the following characters: prosternal basantra strongly developed and longer than wide; pronotal anteromarginal setae minute. Ritchie (1974) provided a key to 18 species of this genus with three new species and four generic synonyms. Subsequently, four species were described from Thailand, Malaysia, China (Taiwan) and India (Bhatti 1978; Okajima 1978), and two from New Zealand (Mound and Walker 1986). Okajima (2006) recorded three species from Japan, Mound and Minaei (2007) recorded 10 species from Australia, and one species was recently described from Iran (Minaei 2015). In China (Taiwan), two species were recorded, *P. lucasseni* (Krüger) and *P. luteus* Okajima (Okajima 1986). At present, the genus *Podothrips* includes 31 species worldwide (ThripsWiki 2019).

As part of ongoing studies on Haplothripini from China, this review of the genus *Podothrips* provides an illustrated identification key to six species with one new species and three newly recorded species.

## Materials and methods

The descriptions, drawings, and photomicrograph images provided here are produced from slide-mounted specimens using an Olympus BX53 and drawing tube. The following abbreviations are used for the pronotal setae:

**am** anteromarginal,

**aa** anteroangular,

**ml** midlateral,

**epim** epimeral,

**pa** posteroangular.

The unit of measurements in this paper is micrometre. Specimens from China, including the holotype of the newly described species, are deposited in the National Zoological Museum of China (**NZMC**) Institute of Zoology, Chinese Academy of Sciences, Beijing, China, with some specimens in the School of Bioscience and Engineering, Shaanxi University of Technology, Hanzhong, China.

## Taxonomy

### Podothrips

Taxon classificationAnimaliaThysanopteraPhlaeothripidae

Hood

5B00C5C5-85FD-50C9-8453-7B3988FF07FC

Podothrips Hood, 1913: 67. Type species: Podothrips semiflavus Hood.

#### Diagnosis.

Small sized, usually bicoloured brown and yellow, but a few uniformly brown. Head smooth, longer than wide, with one pair of postocular setae; antennae eight-segmented, segment III with one or two sense cones, IV with two or three. Pronotum well developed, am always minute; notopleural sutures complete; basantra usually longer than wide; fore tarsus with tooth on inner surface, fore tibia often with a sub-apical tubercle or tooth. Mesopresternum complete, boat-shaped. Metathoracic sternopleural sutures well developed. Forewing fully developed, slightly constricted medially, with or without duplicated cilia. Pelta bell-shaped. Abdominal tergites II–VII each with two pairs of wing-retaining setae. Tube shorter than head, anal setae long than tube.

#### Comments.

This genus is closely related to *Praepodothrips* with which it shares most morphological characters, but it differs in having larger basantra. It is also similar to *Karnyothrips* and *Okajimathrips*, but *Podothrips* can be recognised by the developed basantra and metathoracic sternopleural sutures (*Karnyothrips* species have normal basantra and metathoracic sternopleural sutures absent), and the pronotal notopleural sutures complete (*Okajimathrips* with pronotal notopleural sutures incomplete).

### Key to species from China

**Table:** 

1	Body uniformly brown (Fig. 17)	***P. lucasseni* (Krüger)**
–	Body bicoloured (Figs 18–21)	**2**
2	Prothorax yellow, contrasting with brown head (Fig. 19)	**3**
–	Prothorax brown, concolourous with head (Figs 18, 20, 21)	**4**
3	Abdominal segments I–IX yellow, tube yellow in basal third	***P. luteus* Okajima**
–	Abdominal segments I–VII yellow, VIII–X brown (Fig. 19)	***P. semiflavus* Hood**
4	Metathorax and all femora yellow (Fig. 21)	***P. sasacola* Kurosawa**
–	Metathorax and forefemora brown at minimum	**5**
5	Forewing with duplicated cilia; fore tibia without distinct apical tooth (Fig. 4); most pronotal setae pointed except epim setae expanded (Fig. 4); antennal segment VII brown, concolourous with head (Fig. 18)	***P. odonaspicola* (Kurosawa)**
–	Forewing without duplicated cilia; fore tibia with a distinct apical tooth (Fig. 1); all developed pronotal setae expanded (Fig. 1); antennal segment VII yellow with apical fifth brown (Fig. 20)	***P. femoralis* sp. nov.**

### Podothrips femoralis

Taxon classificationAnimaliaThysanopteraPhlaeothripidae

Dang & Qiao sp. nov.

97EECDDC-A08A-57EE-81E6-279D4690F736

http://zoobank.org/923F14E1-B82B-436B-BF21-6C52B121770C

Figs 1 , 7 , 12 , 13–16 , 20 , 22

#### Female macroptera.

Bicoloured with head, thorax and abdominal segments VIII–X brown, I–VII yellow but III–VII with brown median area; antennal segment I brown, II yellow with brown basal part, III–VII uniform yellow with VI–VII a little darker apex, VIII brown. All legs yellow with fore and middle coxae and fore femora brown (Fig. 20).

**Figures 1–6.**
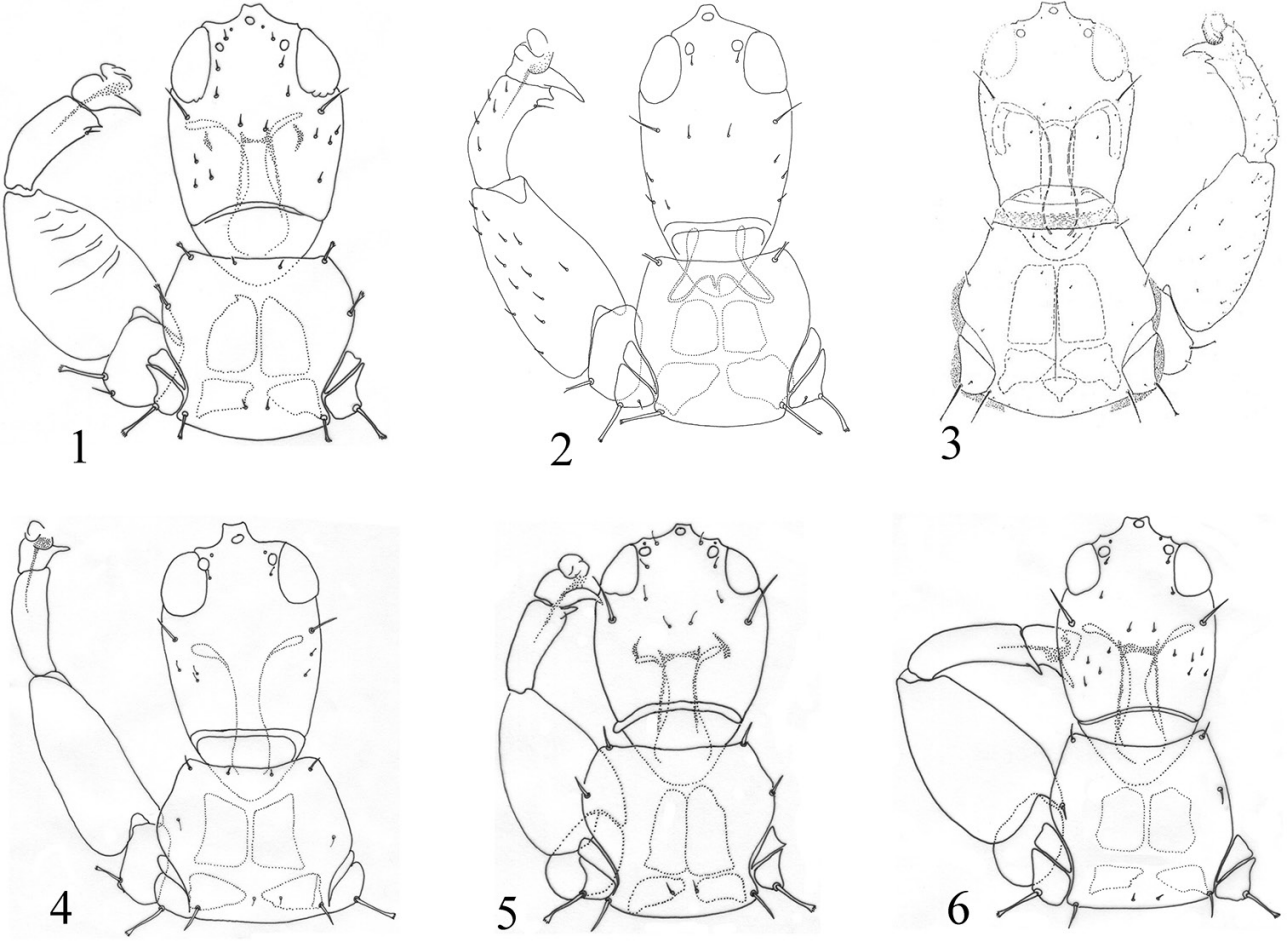
*Podothrips* species head, pronotum& fore legs **1***P. femoralis* sp. nov. **2***P. lucasseni***3***P. luteus* (from Okajima1978) **4***P. odonaspicola***5***P. sasacola***6***P. semiflavus*.

**Figures 7–16.**
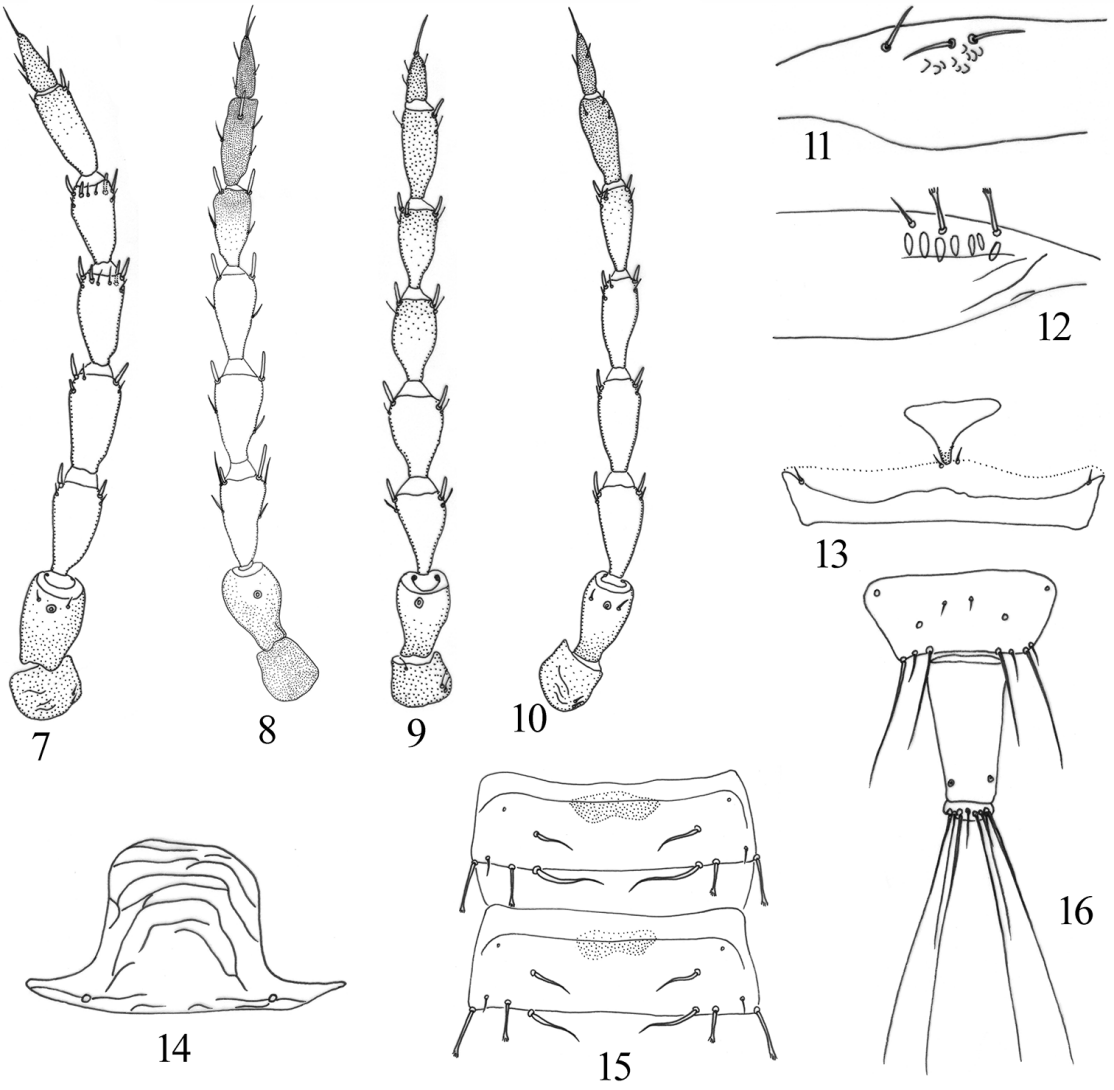
*Podothrips* species. **7–10** Antenna **7***P. femoralis* sp. nov. **8***P. odonaspicola***9***P. sasacola***10***P. semiflavus*. **11, 12** Base of forewing **11***P. sasacola***12***P. femoralis* sp. nov. Some important features of *P. femoralis* sp. nov. **13** mesopresternum **14** Pelta **15** abdominal tergites IV–V **16** abdominal tergites IX–X.

Head 1.2 times as long as wide, cheeks distinctly constricted towards base (Fig. 1); ocellar setae minute; postocular setae pointed at tips, half the length of eye, wide apart from each other (Fig. 1). Mouth-cone short, maxillary stylets reaching base of postocular setae, maxillary bridge present. Antennal segment sense cones: III with 1+1, IV with 1+1^1^, V with 2+2, VI with 1^1^+2, VII with 1 dorsal (Fig. 7).

Pronotum with no sculpture, am reduced, aa, ml, epim, and pa setae well developed with expanded apices, epim setae longest; notopleural sutures complete; basantra well developed, longer than wide (Fig. 1). Metanotum almost smooth; metathoracic sternopleural sutures well developed (Fig. 22). Fore femur expanded; fore tibia with a distinct apical tooth; fore tarsal tooth developed (Fig. 1). Fore wings slightly constricted medially, without duplicated cilia, sub-basal wing setae equal with length, S1 and S2 expanded at apex, S3 acute (Fig. 12).

Pelta hat-shaped with pair of campaniform sensilla (Fig. 14); tergites II–VII with two pairs of wing-retaining setae (Fig. 15); abdominal tergite IX setae S1 and S2 pointed at apex, shorter than tube; tube 0.54 times as long as head; anal setae 1.7 times as long as tube (Fig. 16).

#### Measurements

(holotype female, in µm). Total length 2440. Head length 260, width across behind eyes210; eye length 85, width 55; postocular setae length 40. Antenna length 440, I–VIII length (width): 35(40), 50(30),55(25),60(30),55(25),50(25),52(25),35(22). Pronotum length 235, width 235; aa 12, ml 12, epim 45, pa 17. Fore wing length 960, sub-basal setae S1-S3 length 20, 15, 15. Pelta length 75, width 130.Tube length 140, anal setae length 240.

#### Specimens examined.

Holotype female. CHINA, Yunnan, Mengla County, on Bamboo leaves, 22.iv.1997, Y.F. Han. Paratype: one female with same data as holotype; one female, Fujian Prov., Xiamen City, on Bamboo leaves, 29.iv.1991, Y.F. Han; one female, Guangdong Prov., on Bamboo leaves, 29.iv.1992, Y.F. Han.

#### Comments.

This new species is similar to *P. sasacola* in forewing without duplicated cilia and body bicoloured, but differs in having all legs yellow with fore legs femora brown, antennal segment V–VI uniformly yellow and VII yellow with apical third brown (Fig. 20), meso- and metanotum brown, all developed pronotal setae expanded at apex (Fig. 1), and fore wing sub-basal setae S1 and S2 expanded (Fig. 12). In contrast, *P. sasacola* has all legs yellow, antennal segments V–VI yellow with apical half brown, VII uniformly brown, meso- and metanotum yellow (Fig. 21), pronotum aa, ml and pa pointed at apex, epim setae expanded (Fig. 5), and sub-basal setae S1 and S2 pointed (Fig. 11). It is also related to *P. odonaspicola* and *P. bicolor* Seshadri & Ananthakrishnan in the bicoloured body, but this new species can be distinguished by forewing without duplicated cilia (forewing with duplicated cilia in *P. odonaspicola*), and fore tibia with distinct subapical tooth (fore tibia without distinct subapical tooth in *P. odonaspicola*) (Figs 1, 4), and by fore femora brown (all femora yellow in *P. bicolor*).

#### Etymology.

This species name is composed of one Latin word, *femoralis*, based on the brown fore femora.

### Podothrips lucasseni

Taxon classificationAnimaliaThysanopteraPhlaeothripidae

(Krüger)

93A266F9-C824-5B26-8E46-C0FBA4432D65

Figs 2 , 17

Phlaeothrips lucasseni Krüger, 1890: 105.

#### Remarks.

**Figures 17–22.**
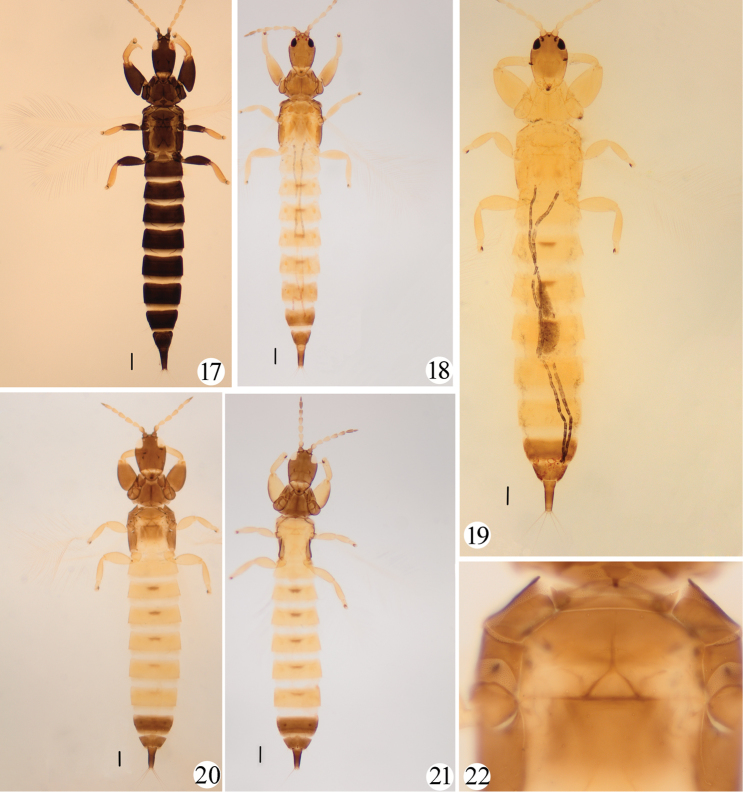
*Podothrips* adult colour patterns **17***P. lucasseni***18***P. odonaspicola***19***P. semiflavus***20***P. femoralis* sp. nov. **21***P. sasacola*. Some important features of *femoralis* sp. nov. **22** mesopresternum and metathoracic sternopleural sutures. Scale bars: 200 microns.

Described from Java on sugar cane, and widely distributed in Asia, this is the only *Podothrips* from China that is uniformly brown (Fig. 17). *P. hawaiiensis* from Hawaii and *P. oryzae* from Thailand were placed as synonyms of *P. lucasseni* by Ritchie (1974). This species was recorded by Okajima (1986) from China (Taiwan), and a female and a male from Guizhou Province have been examined here.

### Podothrips luteus

Taxon classificationAnimaliaThysanopteraPhlaeothripidae

Okajima

BB80DCE1-E5A4-557D-8A94-0A77EA75A575

Fig. 3

Podothrips luteus Okajima, 1978: 34.

#### Remarks.

This species is known only from China (Taiwan) on grass. Unfortunately, no specimens were examined here. According to the description, it can be distinguished easily from the other species considered here by the bicoloured body with most of the abdomen yellow – abdominal segments I–IX and basal third of tube yellow (Okajima 1978).

### Podothrips odonaspicola

Taxon classificationAnimaliaThysanopteraPhlaeothripidae

(Kurosawa)

926F6C41-AF7A-5FE4-BB44-4C714CA3257C

Figs 4 , 8 , 18

Haplothrips odonaspicola Kurosawa, 1937: 266.

#### Remarks.

Described from Japan (Tokyo) on bamboo leaf sheaths, this species is recorded here from China (Sichuan, Hubei) for the first time, based on three females. The brown thorax and yellow abdominal pattern are similar to the new species, *P. femoralis*, but it may be distinguished by the forewing with duplicated cilia and fore tibia without distinct subapical tooth (Fig. 4).

### Podothrips sasacola

Taxon classificationAnimaliaThysanopteraPhlaeothripidae

Kurosawa

16AAD34F-3D3B-5F5E-B354-9BC5E2594605

Figs 5 , 9 , 11 , 21

Podothrips sasacola Kurosawa, 1940: 100.

#### Remarks.

Previously known only from Japan, this species is quite similar to *P. bicolor* in the body colour pattern – head, pronotum, and abdominal segments VIII–X brown. Specimens are identified here as *P. sasacola* have antennal segments III–IV each with two sense cones (Fig. 9), and the fore tibia with a distinct inner apical tubercle (Fig. 5) as described by Okajima (2006). This species is recorded here for the first time from China, Sichuan, based on five males taken from reeds.

### Podothrips semiflavus

Taxon classificationAnimaliaThysanopteraPhlaeothripidae

Hood

7D1F1624-1589-5205-82BA-A9C757D9938F

Figs 6 , 10 , 19

Podothrips semiflavus Hood, 1913: 67.

#### Remarks.

Described from Puerto Rico, America on *Panicum* leaves, this species is recorded from Egypt and Uganda by Ritchie (1974), with *P. aegyptiacus* Priesner placed as a synonym. This is one of two species from China in which the thorax is yellow (Fig. 19), but *P. luteus* from Taiwan has abdominal segments VIII–X brown, whereas the abdomen of *P. semiflavus* is almost yellow with just the basal third of the tube brown (Fig. 19). One female from Guangdong has been studied here, and this is the first record of the species from China.

## Supplementary Material

XML Treatment for Podothrips

XML Treatment for Podothrips femoralis

XML Treatment for Podothrips lucasseni

XML Treatment for Podothrips luteus

XML Treatment for Podothrips odonaspicola

XML Treatment for Podothrips sasacola

XML Treatment for Podothrips semiflavus
